# The effect of long-term exposure to microgravity on the perception of upright

**DOI:** 10.1038/s41526-016-0005-5

**Published:** 2017-01-12

**Authors:** Laurence R. Harris, Michael Jenkin, Heather Jenkin, James E. Zacher, Richard T. Dyde

**Affiliations:** Centre for Vision Research, York University, 4700 Keele St., Toronto, ON M3J 1P3 Canada

## Abstract

Going into space is a disorienting experience. Many studies have looked at sensory functioning in space but the multisensory basis of orientation has not been systematically investigated. Here, we assess how prolonged exposure to microgravity affects the relative weighting of visual, gravity, and idiotropic cues to perceived orientation. We separated visual, body, and gravity (when present) cues to perceived orientation before, during, and after long-term exposure to microgravity during the missions of seven astronauts on the International Space Station (mean duration 168 days) and measuring perceived vertical using the subjective visual vertical and the perceptual upright. The relative influence of each cue and the variance of their judgments were measured. Fourteen ground-based control participants performed comparable measurements over a similar period. The variance of astronauts’ subjective visual vertical judgments in the absence of visual cues was significantly larger immediately upon return to earth than before flight. Astronauts’ perceptual upright demonstrated a reduced reliance on visual cues upon arrival on orbit that re-appeared long after returning to earth. For earth-bound controls, the contributions of body, gravity, and vision remained constant throughout the year-long testing period. This is the first multisensory study of orientation behavior in space and the first demonstration of long-term perceptual changes that persist after returning to earth. Astronauts showed a plasticity in the weighting of perceptual cues to orientation that could form the basis for future countermeasures.

## Sensory perception: Knowing which way is ‘up’

Prolonged exposure to microgravity has a long-term effect on the perception of upright. On earth we use visual, body, and gravity cues to help us determine the orientation of ourselves relative to the world which affects many perceptual tasks including reading, recognizing faces, and navigating. Laurence R. Harris and colleagues at York University assessed how seven astronauts who spent 168 days on average on the International Space Station perceived their orientation before, during and after flight. Although no changes were observed during their missions, astronauts’ judgements in the absence of visual cues were worse upon return to earth compared with ground-based controls. Harris and his team found that the effect persisted for up to four months after the astronauts returned to earth. These findings could help develop countermeasures to avoid perceptual mistakes during space travel, and contribute to facilitating safer, long-duration journeys without gravity.

## Introduction

Humans have evolved in the gravitational field of the earth that provides a constant direction to which spatial perceptions and movements can be referenced. Deriving the direction of gravity from sensory information is not straightforward. Although the otoliths of the vestibular system are sensitive to gravity’s effects their signal is ambiguous as the vestibular system cannot on its own distinguish gravity from other accelerations. The vestibular system therefore needs to be constantly calibrated, normally relying on visual and somatosensory cues. The midline of the body also contributes, acting as an idiotropic prior that assumes the body is upright^1,2^ and to which the perception of upright tends to revert in the absence of other cues. Under normal circumstances gravity, body and vision cues all contribute to the estimation of vertical and are weighted in proportion to their reliability.^3^ The relative weighting varies between participants and between tasks, presumably reflecting individual factors such as the stability of the eyes, the reliability of the internal representation of the body, and the efficiency of the vestibulo-somatosensory system. How is this multisensory system affected when gravity is removed? Do visual cues remain as effective in determining the perception of “up” during and after long-term exposure to microgravity?

When microgravity is created for short periods of time using parabolic flight, the weightings of the remaining vision and body cues do not remain constant. There is a tendency for even clearly visible orientation cues to be less strongly weighted, and for a person’s assessment of vertical to revert towards the internal idiotropic vector.^4^ However, confounds in short-duration microgravity experiments include the dynamic changes in the external forces acting on the body and the lack of time for any substantial adaptation. Understanding how microgravity affects human perception is critical for the development of safe, long-duration space travel. A substantial body of work has revealed that perception is indeed altered in long-duration microgravity. Visual re-orientation illusions, inversion illusions, and motion sickness are regularly reported in weightlessness.^5,6^ (see^7^ for a review) Experiments performed during and immediately after space flight have shown that perception of self-orientation is altered^8,9^ and that tilt of the body is overestimated immediately on return to earth.^10–13^ Not only are many of these effects debilitating, but they also represent a serious safety hazard when navigating in an emergency or when operating oriented switches. In order to deal with this it has been proposed that “visual gravity” should be introduced into space craft where visual cues to orientation are generally ambiguous.^14^

Unfortunately for space travelers, the effects of long-duration spaceflight on perceptual and physiological systems also influences re-adaptation to gravity on return to earth (see^15^ for a review), which is a concern for interplanetary missions in which astronauts landing on other planets must function without support of a ground-based recovery team. “How long does it take for astronauts to recover from perceptual and physiological adaptations associated with long-duration spaceflight?” is a fundamental question that must be answered before we can venture to other planets and asteroids safely.

There are several ways to measure the perceived vertical, and the relative weighting of the cues involved vary with the measure chosen.^3,16^ In this study we chose the subjective visual vertical (SVV)^17^ and the perceptual upright (PU).^3^ The decision of which probes to use was based on several factors. We required a test that was sensitive to the controlled visual cues that we could provide on orbit, and practical concerns put severe constraints on the amount of astronaut time that could be allocated to the project. Vision has only a small influence on the SVV^3^ and since the SVV probe involves judging the orientation of a line relative to gravity, it cannot sensibly be used in microgravity (but see^18^). The PU measures the orientation at which objects and characters are most easily recognized, a perceptual correlate of the perceived direction of up, and is measured by the Oriented Character Recognition Test (OCHART).^3^ OCHART has been used successfully on earth,^3^ during parabolic flight,^4^ and in studies with Parkinson’s populations.^19^ Given the success of previous experiments using the OCHART probe with both experienced and naïve observers, its usability in microgravity, and the relatively equal influence of gravity, body and visual cues upon it, OCHART was selected as our test of choice for the in-flight experiments, and both SVV (measured using a luminous line) and PU (measured by OCHART) were measured before and after flight. In order to control for repeating the tests multiple times, the SVV and PU were also tested on a control group of naïve participants over approximately the same period of time as the astronaut testing.

The perceived direction of gravity is determined by a combination of the directions signaled by the long-axis of the body, gravity, and visual cues in which the weightings allocated to each cue by the central nervous system can be inferred using a statistical approach.^3^ The purpose of this study was to examine how the weightings applied to each cue might vary during and after spaceflight. To do this we assessed astronauts before, during, and after their missions on the International Space Station and compared their performance with that of ground-based controls measured over a comparable period. Testing sessions on earth are referred to as baseline data collection sessions (BDC1 was carried out before flight, BDC2 and BDC3 after flight) and in-flight sessions early and late in-flight are referred to as FLTE and FLTL respectively. Our hypotheses were that (i) astronauts’ subjective visual vertical and perceptual upright would be impacted by long duration spaceflight, (ii) that the weighting of visual cues relative to body cues would be reduced during spaceflight in estimating the direction of up as measured by the effect of vision on the PU, and (iii) that all weightings would return to preflight values within a few weeks of return to earth.

## Results

### Subjective visual vertical

**Fig. 1:**
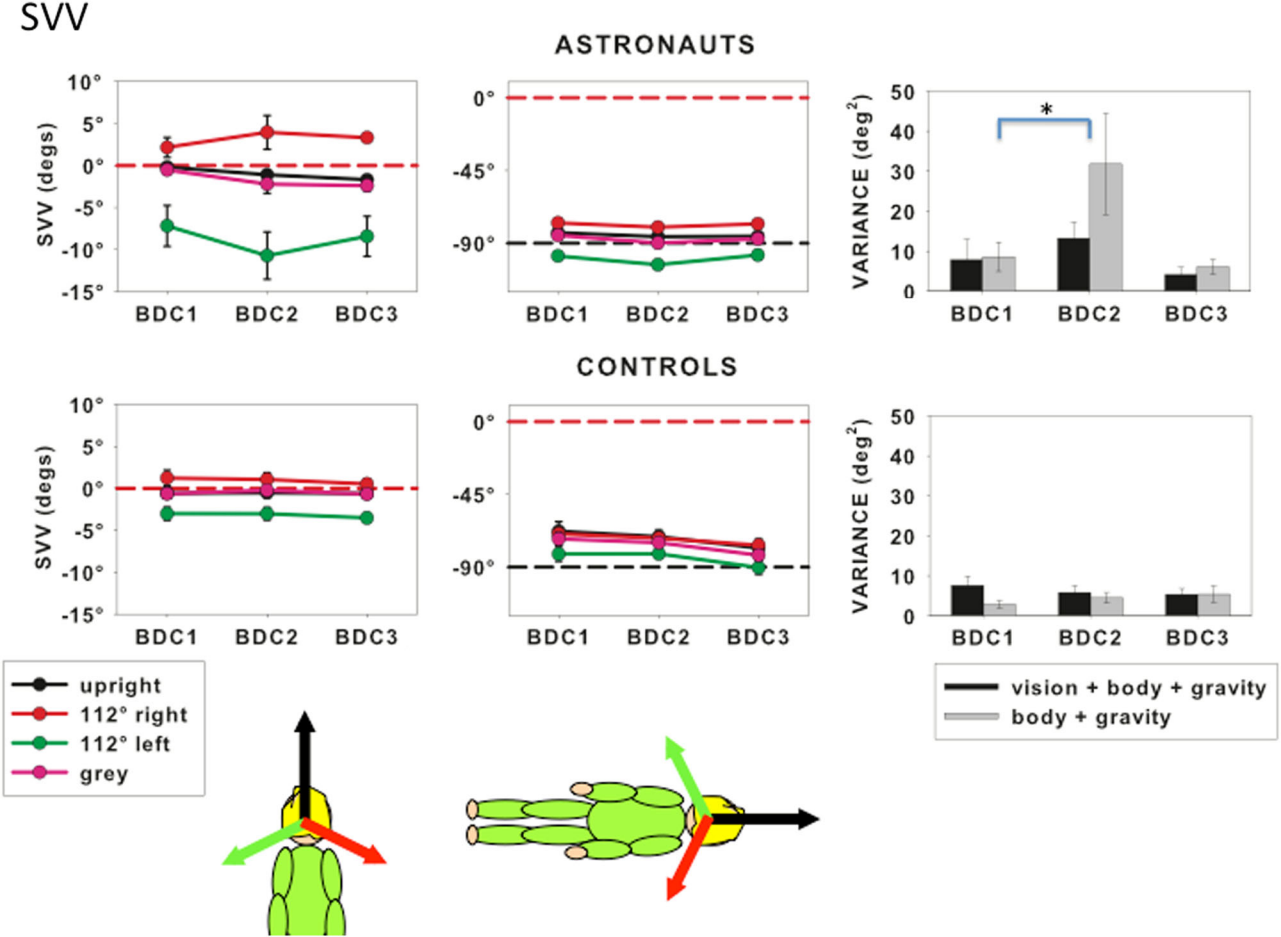
The SVV for astronauts (*top row*) and control participants (*bottom row*) for BDC1 (before flight), BDC2 and BDC3 (after flight). 0° corresponds to the top of the head (*red dashed lines*), negative values indicate tilt to the *left*. Means and standard errors across participants are shown for the SVV measured in the presence of four visual backgrounds: upright (*black lines* and *symbols*), tilted to right (*red lines* and *symbols*), tilted to *left* (*green lines* and *symbols*), and *gray* (*purple lines* and *symbols*). *Inserts* show the direction of the visual cues relative to the body. The histograms show variances in deg^2^ for the upright SVV with (*black bars*) and without (*gray bars*) visual cues to upright. *Asterisks* indicate that variances for BDC1 were significantly lower than for BDC2 when only body and gravity cues contributed

The SVV for earth-based controls and astronauts, measured upright or right side down with visual cues in the directions shown or against a gray background for the three BDCs is shown in Fig. 1. The average period between BDC1 and BDC3 was 330 days for the control group and 405 days for the astronauts. When upright and viewing the line probe against a tilted background, the SVV was shifted in the direction of the visual cue by 2.6° ± 0.9° for controls and by 6° ± 3.6° for astronauts. When participants lay on their right side, the SVV was tilted by −73° ± 4° for controls and −81° ± 4° for astronauts. That is, the SVV remained close to the gravitational vertical (−90° when lying right side down). These data confirm previous observations that the SVV is dominated by the gravity cue and that vision plays a small, although significant role. Repeated measures ANOVAs were performed separately for the controls and astronauts in both right-side-down and upright conditions. No statistically significant effect of collection session was found for either group in either posture.

The variances in the SVV measurements are shown on the right of Fig. 1 for conditions in which all the cues were upright. No effect of BDC session was found for controls, however a significant effect of BDC session was found for astronauts *F*(2, 12) = 5.306, *p* = 0.022. Post-hoc analysis using 1-tailed *t* tests and Bonferroni correction indicated that BDC2 (31.8 ± 12.7 deg^2^) had significantly greater variance than BDC1 (8.6 ± 3.6 deg^2^) (*t*(6) = 2.44, *p* = 0.05). Astronauts were more uncertain in their SVV judgments immediately upon return to earth (BDC2) compared to their preflight performance (BDC1). Their level of certainty returned to preflight levels by BDC3.

### Perceptual upright

**Fig. 2:**
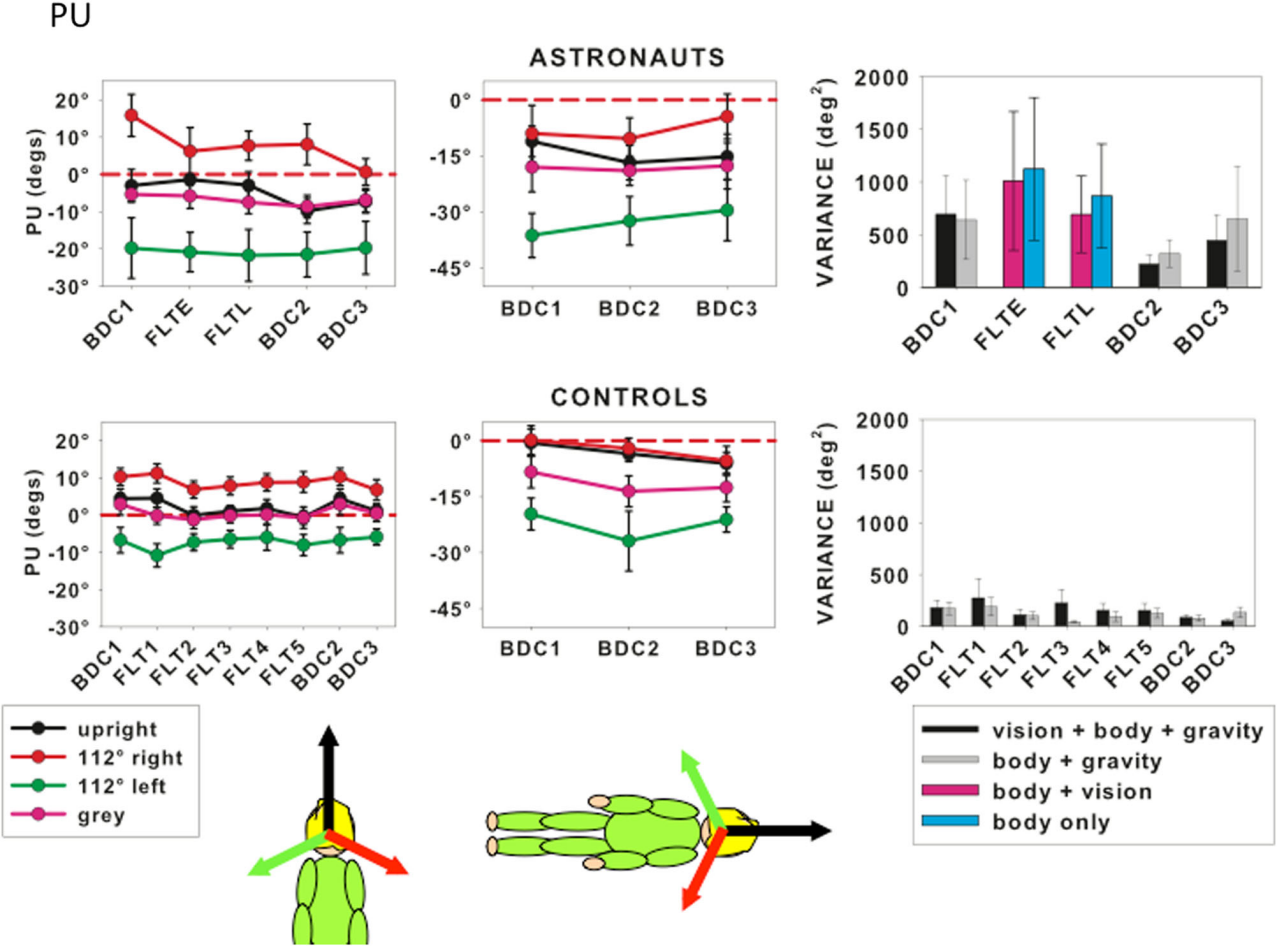
The perceptual upright for astronauts (*top row*) and control participants (*bottom row*). Astronaut data from early (FLTE) and late in flight (FLTL) are shown together with the three BDC measurements. Right-side-down experiments (*middle column*) were only performed in the BDC sessions. Format as for Fig. 1. Variances for the in-flight measures correspond to “body only” when performed against a *gray* background (*pale blue*) and “body + vision” when upright visual cues were provided (*purple bars*)

The PU for earth-based controls and astronauts measured upright or right side down with visual cues in the directions shown or against a gray background, is shown in Fig. 2 for the three BDCs and FLT trials. For the astronauts, only early in flight (FLTE) and late in flight (FLTL) in-flight data are shown (but see next section) because these were the only trials in which all astronauts participated. When participants were tested lying on their right side against a gray background, the PU was tilted by −8.4° ± 4.3° for controls and −18° ± 6.6° for astronauts. That is, the PU remained relatively close to the body vertical (0°) when lying right side down with a relatively minor effect of gravity. No statistically significant effect of collection session was found for astronauts or controls in either upright or right-side-down body postures for the PU or the associated variances.

#### Voluntary science

**Fig. 3:**
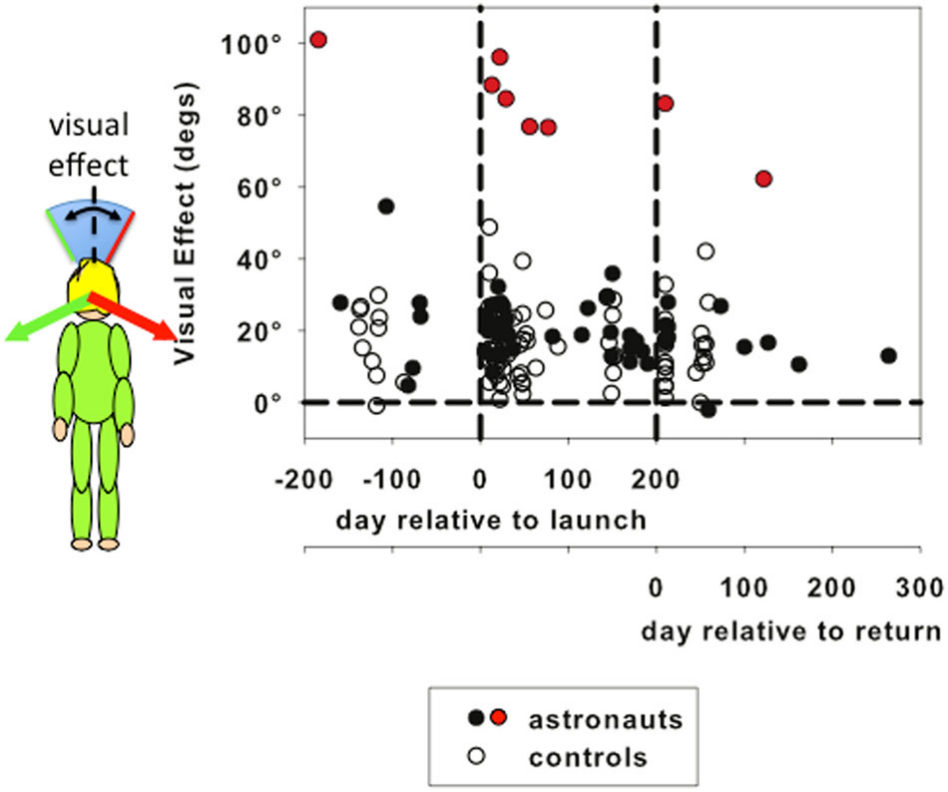
The visual effect (VE) (shown as the *blue shaded area* in the insert) plotted as a function of time since launch and of the day relative to return to earth with each observer’s data plotted separately. *Data* from every session performed are plotted for ground controls (*open circles*) and astronauts (*filled circles*). One astronaut (*filled red circles*) had a substantially larger VE than the others. Details of the time of each data collection session are given Table S2

Several astronauts were able to perform the PU experiment in space more often than just at the beginning and end of their mission. To summarize the influence of vision on the perceptual upright a convenient measure is to take the difference between the PU with the background tilted equally left and right (see insert to Fig. 3). This is referred to as the “visual effect” (VE).^3^ The VE for both the controls and astronauts is plotted in Fig. 3 as a function of time since launch (or simulated launch) and time after return (or simulated return). Although the visual effect hovers around 20° for most sessions in both groups, one astronaut (shown as red symbols) had a substantially larger VE (averaging around 85°). This is not unusual in the normal population^3^ but the high values influence the means plotted in Fig. 2. Given the high degree of variability in the astronaut pool and its relatively small sample size, non-parametric statistical testing is used in the modeling below.

#### The weighted vector model

**Fig. 4:**
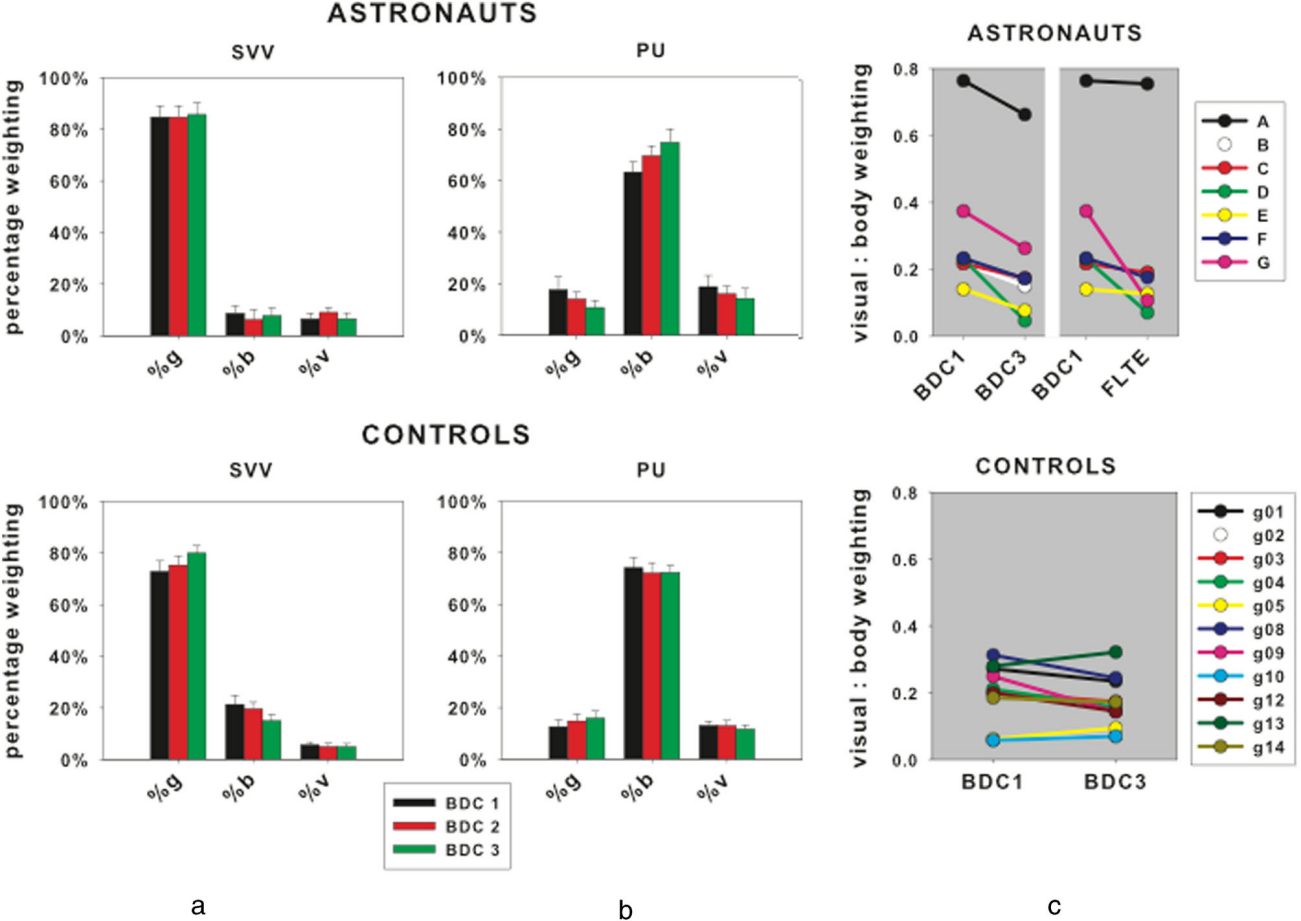
The weightings of the three cues (gravity, body and vision) that determine the SVV (**a**) and PU (**b**), expressed as percentages, for astronauts (*top row*) and controls (*bottom row*) for each BDC session. SEs are also shown. **c** shows the ratio of vision to body weightings for each observer taken from the PU analysis for BDC1, FLTE and BDC3 for astronauts, and BDC1 and BDC3 for controls

The VE did not vary significantly in response to spaceflight (or for ground controls) but the null result on going into space was actually surprising. We can model both the PU and SVV as being determined by a combination of gravity, body, and visual cues as a linear weighted sum of three vectors pointing in the directions signaled by each cue^3^ as:

1 $${\rm{up}}=\, {\rm{vision}}\ast {{\rm{weight}}}_{{\rm{vision}}}+{\rm{body}}\ast {{\rm{weight}}}_{{\rm{body}}} \\ +{\rm{gravity}}\ast {{\rm{weight}}}_{{\rm{gravity}}}+\mathrm{bias},$$

where vision, body, and gravity are vectors associated with each cue, each with its own weighting expressed relative to the others. This model has proven adequate to explain a number of cue integration results^20^ although more sophisticated models exist.^21^ In our experiments, the directions indicated by each cue were separated so that the relative magnitudes of the weights could be calculated. Data obtained using the SVV and PU probes in the upright and right-side-down conditions and with different visual backgrounds were used to fit Eq. 1 using a least squares non-linear optimization process (see Supplemental Material for details) and to quantify the relative weighting of vision, gravity and body cues contributing to the SVV and PU for each BDC (Fig. 4). A similar method was used to obtain the relative weightings of vision and the body contributing to the PU during the in-flight conditions in which the direction of vision was varied relative to the body.

Although Friedman tests showed that the weightings for SVV and PU data did not change across BDC session for either the astronauts or the controls (Fig. 4a, b), there was a trend in the astronauts’ PU weightings for an increased body weighting (from 63 to 75%) and a corresponding decrease in visual weighting (from 19 to 14%) across BDC sessions. 100% of astronauts showed a reduction in the ratio of the vision to body weightings (v:b) for the PU measurements from BDC1 to FLTE and from BDC1 to BDC3 (Fig. 4c). These reductions were confirmed as significant using Wilcoxon signed-rank tests (*Z* = −2.366, *p* = 0.018 for both). Given the difference in response between astronaut A and the remainder of the astronauts, the analysis was repeated using only astronauts B–G. The reductions remained significant, albeit with higher *p* values (*Z* = −2.201, *p* = 0.028 for both). No comparable effect was found for the ground-based controls (Fig 4c). The v-b ratios of the control and astronaut groups in the baseline (BDC1) condition were not significantly different therefore initial group differences cannot account for the differences between the astronaut and control groups across the different measurement sessions.

**Fig. 5:**
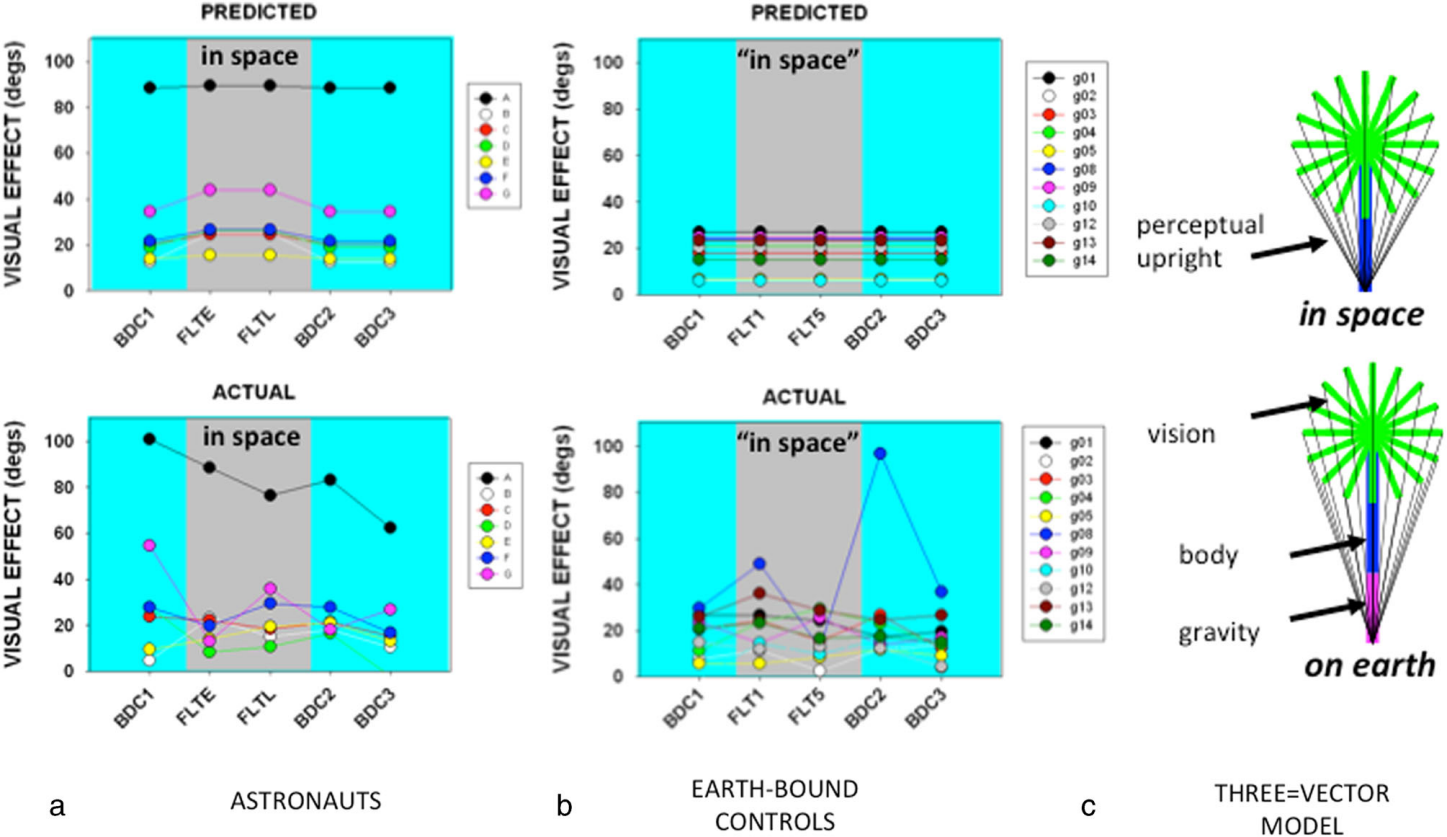
The size of the visual effect for each astronaut (**a**, *lower panel*) and ground control (**b**, *lower panel*) is compared to the effect that would be predicted if the relative weights of vision and body remained constant (*upper panels*). The *gray* shaded region indicates the measurements taken in space (or at equivalent times for the controls). FLTE and FLTL refer to measurements taken early (9–14 days) and late (77–190 days) in flight, or at the equivalent times (FLT 1 and FLT 5) for the controls. The three-vector model is shown diagrammatically in **c** to illustrate the predicted effect of removing the gravity cue (*top*)

When an observer lies supine on earth the influence of gravity is removed from the long-axis of the body and therefore, because of simple geometry (adding two vectors instead of three), the visual effect should increase. This predicted increase occurs reliably on earth.^3^ Exposure to microgravity should increase the visual effect in the same way. Figure 5 shows this prediction for each astronaut (Fig. 5a) and control (Fig. 5b) calculated assuming that the relative weights assigned to the vision and body cues (determined in BDC1) remain constant (Fig. 5c). Under this model the three BDCs are predicted to have the same visual effect, and the visual effect is expected to increase when gravity is removed. However, the visual effect did not show such an increase when measured early in flight (FLTE) (Fig. 5a), and long after returning to earth (BDC3) astronauts’ visual effects were significantly less than would be predicted if the weightings had remained constant (*Z* = −2.366, *p* = 0.018). The visual effect remaining constant on arrival in space is consistent with the reduction in v-b ratio that occurs at that time such that the missing gravity component is replaced with a relative increase in the weighting applied to the body cue (Fig. 4c). The reduction after return (BDC 3) would then be because the relative increase in body weighting is inappropriate once the gravity cue is again contributing (Fig. 4c). A series of Wilcoxon signed-rank tests showed that ground controls (Fig. 5b) exhibited no such changes.

#### Are the variances consistent with the weightings?

**Fig. 6:**
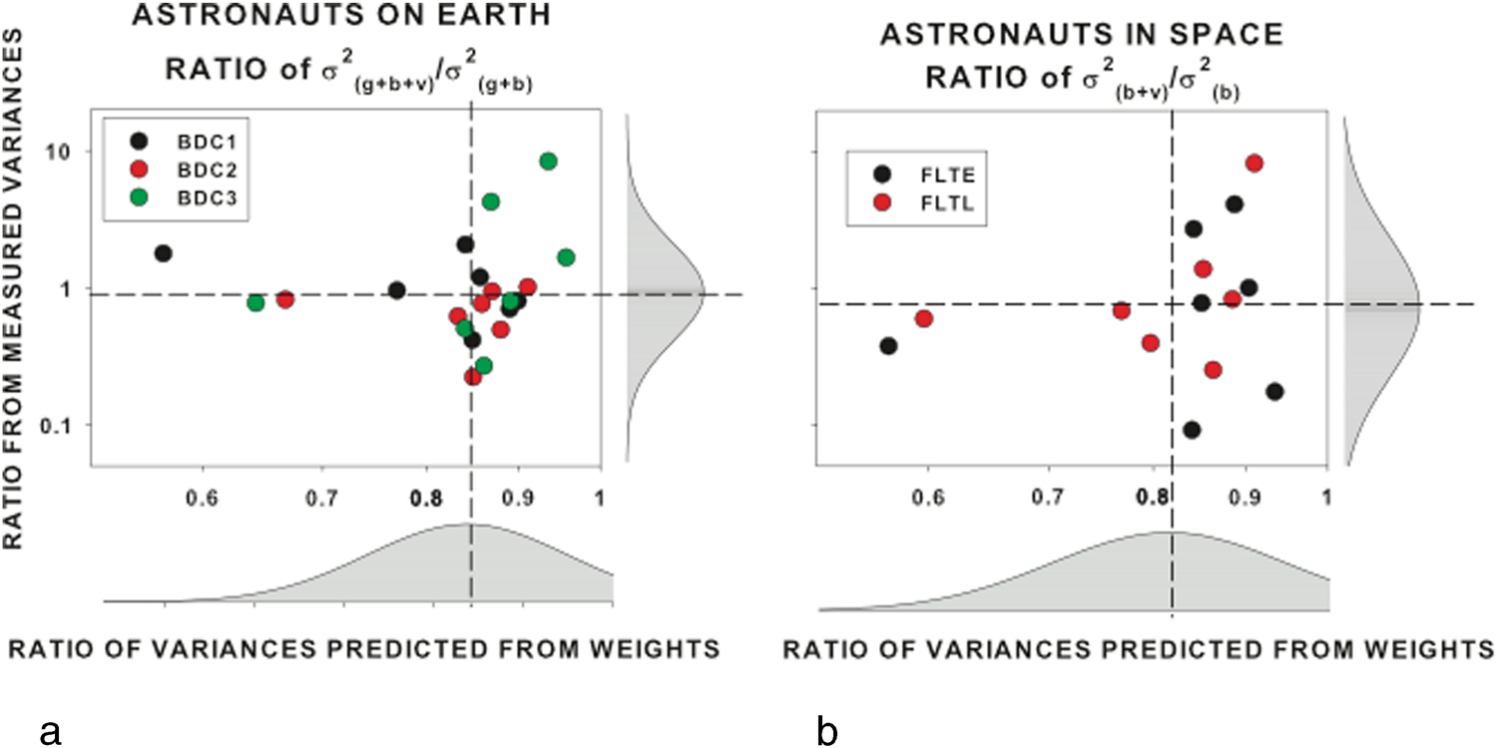
Comparison of variances calculated from the weightings using the vector sum model (see text, *horizontal axis*) with the measured variances (*vertical axis*). Each axis plots the ratio of variance with all cues present to the variance without the visual cue on log–log scales. **a** On earth, gravity, body and vision cues contribute to the all-cues-present condition. **b** In space, only body and vision cues are present. Distributions of the data are given on each axis

According to maximum likelihood theory, the weights assigned to the various cues should be inversely proportional to the variance of each cue. Although our assessments of the variances were too noisy to calculate the expected weightings (see Figs 1 and 2), we can work backwards to calculate the expected ratio between the variances. The weightings for the BDC data were obtained as indicated above and for the in-flight data the relative weights of vision and the body were calculated geometrically from the VE (see Fig. 5c). Assuming that the weights are inversely proportional to the variance $({w}_{v}=k/{\sigma }_{v}^{2})$ and that the constants of proportionality are constant, we can obtain an estimate of relative variances in each condition. For example, ${\sigma }_{{\rm{b}}+{\rm{v}}}^{2}=k/((k/{\sigma }_{{\rm{b}}}^{2})+(k/{\sigma }_{{\rm{v}}}^{2}))=k/({w}_{{\rm{b}}}+{w}_{{\rm{v}}})$. Without the constant of proportionality (*k*) the “*k*-variances” can only be expressed as ratios of each other. The *k*-variance expected, when all the cues are present was calculated from the weights in this way and expressed as a ratio of the *k*-variance when only gravity and body cues were present (with a gray background) for each astronaut in each BDC session (mean 0.84 ± 0.1). The ratio between the *k*-variances when vision and body were the only cues available (in space; FLTE and FLTL) was similarly expressed as a ratio of the *k*-variance when the body was the only cue available in space (0.89 ± 0.50). In each case, the variance should be reduced when more cues are available and so these ratios are expected to be less than one. The ratios calculated from the weights in this manner are plotted against the comparable ratios of the actual measured variances in Fig. 6 for all astronauts in each data collection session. The ratios obtained from the k-variances were not significantly different from the ratios of the measured variances for either comparison. This indicates that the relationship between the reliability of each cue and its weighting is consistent with MLE principles.

## Discussion

For both ground-based and astronaut participants, the SVV and PU responses remained consistent across data collection sessions even though astronaut observers were subjected to microgravity for an average of 168 days (Figs 1 and 2). The variance of the SVV measured without visual cues while upright immediately after return to earth (BDC2) was significantly larger than before flight (BDC1) but returned to preflight levels by BDC3 (Fig. 1). Modeling individual PU responses as a weighted linear vector sum revealed a significant reduction in visual relative to body weighting between BDC1 and on first arriving in space (FLTE). This reduction was also found in BDC3, on average 130 days after return to earth (Fig. 5c).

### No change in visual effect on arrival in space

The stability in the visual effect on first going into space contrasts sharply with earlier results using transient periods of microgravity created by parabolic flight^4^ in which an instant reduction in visual weighting was found. Here, we demonstrate that astronauts adapt within the first 10 days of flight (during which time we unfortunately did not have access to them) to their new perceptual environment such that the influence of vision in determining their perceptual upright was comparable to the visual influence they showed on earth prior to their space mission. The lack of the expected increase in visual effect for the FLTE data suggests that in space the body cue is given more weight relative to vision than it normally has on earth. It is important to note, however, that when tested in space astronauts held on to the COGNI tunnel that was attached to the ISS, which provides a tactile frame of reference. While this was the same arrangement as used in ground-based controls, it may be that tactile cues are more influential in space^22,23^ which may have contributed to strengthening the influence of the body cue.^24^ However this increase is achieved, it appears that astronauts adapt to a microgravity environment by adjusting the relative weights given to the visual and body cues, thus canceling the increase in visual influence on orientation that would be expected if the weights had retained their on-earth values. A tendency to rely more on the body cue for orientation has been seen in earlier microgravity experiments^9^ but has never before been quantified. In contrast to this reduced dependence on static visual cues for orientation, dynamic visual cues seem to be more effective in space, for example in evoking vection.^25,26^ However, these observations may be connected to misperception of static cues to distance in microgravity.^27,28^ We are currently engaged in further space-based experiments to resolve these apparent differences.

### Effects persist after return to earth

There were no significant changes in the direction or influence of vision on the SVV following spaceflight, although it is possible there were changes within the first few days after return when we did not have access to the astronauts. An unexpected finding, however, was that when the astronauts were tested many days after return (average 130 days, range 68–285 days), there was a significant reduction in the weighting placed on the visual cue relative to the body cue (v:b) in determining the PU, relative to BDC1. The reduction following spaceflight was comparable to the reduction we observed on first going into space (Fig. 4c). If a decline in emphasis on vision were part of adapting to microgravity, as we postulated to explain the results found in short-duration microgravity,^4^ then we might expect to see the reduction in the ratio of the weighting of vision relative to body to persist for some time upon return to earth. The ratio of vision to body weighting at BDC2 was unchanged relative to BDC1, but decreased over the next few months when BDC3 measurements were taken. What could have caused this long-term change and why was it not seen immediately on landing? It has been known anecdotally for some time that going into space can affect vision, especially near acuity. John Glenn actually kept a pair of “space anticipation glasses” onboard his capsule. Reports are emerging of visual deterioration during and following space flight^29,30^ although the details are still not fully understood. It is possible therefore that the reduction in weighting of the visual cue we noticed in space is attributable to an actual deterioration of vision. Such an explanation, however, would predict an increase in variance for tasks involving vision that was not observed (upright-with-vision variances for our astronauts between BDC1, 695 ± 363 deg^2^ and BDC3, 446 ± 243 deg^2^). Also, such visual deterioration is most pronounced during microgravity exposure and seems to show good recovery after returning to earth^29^ (see also Chris Hadfield’s anecdotal reports (The web abounds with Chris Hadfield anecdotes, see for example http://globalnews.ca/news/568008/chris-hadfield-after-space-a-windy-road-to-health-recovery/ and some descriptions in his popular book “An astronaut’s guide to life on earth”)). Another possibility is the phenomenon of flashback—astronauts may suddenly and unexpectedly feel that they are back in space long after they are actually safely on the ground. A similar phenomenon is well-documented in virtual reality users^31,32^ and may have been evoked in our astronauts, when they used the equipment that they were familiar with in space. While these speculations are feasible hypotheses, our data cannot specifically address them. Our finding of decreased weighting of vision a few months postflight was unexpected and warrants further investigation.

### SVV variance increased upon return to earth

A significant increase in variance associated with the SVV in the absence of visual cues was found for upright astronauts upon return to earth (BDC2) relative to preflight (BDC1). The SVV requires participants to judge the direction of gravity explicitly, something that astronauts would not have been able to do while in space. This lack of experience may have contributed to astronauts’ difficulty in judging the SVV shortly after returning to earth. Lack of precision in judging the direction of gravity may be related to postural and gait instabilities associated with return from spaceflight.^15^

### The effect of previous space experience

As a group, our seven astronauts had relatively little experience in space prior to their participation in our study. Although four out of the seven had been to space before, only one had prolonged space experience (astronaut E, 193 days). Astronaut E also had the lowest visual weighting (11%) out of our astronaut population prior to launch (BDC1), which was maintained through till our final testing long after returning to earth (BDC3) (Fig. 4a). Of course we are unable to say if this single astronaut’s low visual weighting was caused by previous space experience, but the observation is compatible with the idea that a reduced weight given to vision could be a permanent consequence of space travel as has been proposed after short-duration spaceflight,^8^ parabolic flight^4,33^ and during in-flight centrifugation^34^ (see^15^ for a review).

### The effect of age

**Fig. 7:**
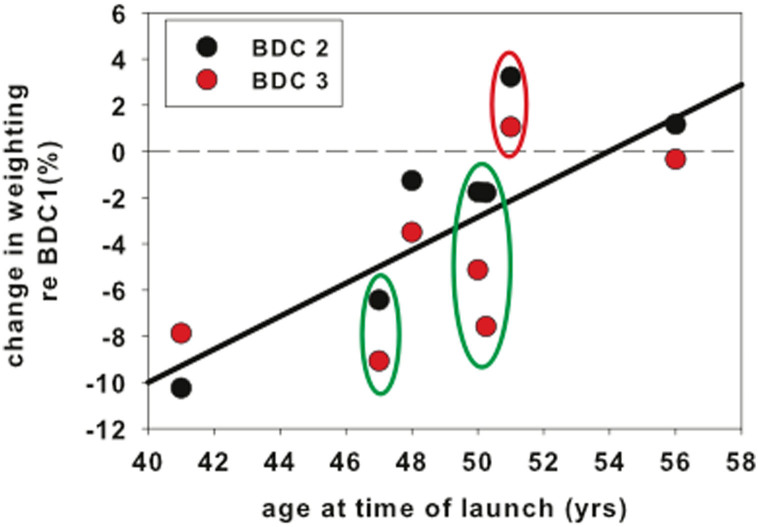
The change in visual weighting (relative to BDC1) is plotted as a function of age-at-launch for BDC2 and BDC3 data. Positive changes correspond to an increase in visual weighting. The regression line though the data has an *r* ^2^ of 0.55. The data *circled* in *red* are from astronaut E who had 193 days of space experience prior to launch, and the data *circled* in *green* are from astronauts with no prior space experience

Our astronauts’ ages spanned from 41 to 56 years at time-of-launch. Previous studies have suggested that there might be an effect of age on the ability to use gravity in the perception of orientation^35^ and certainly on susceptibility to visual reorientation illusions^36^ suggesting progressively less reliance on gravity as one gets older. Although ours was a small population, Fig. 7 plots the reduction in visual weighting between BDC1 and BDC2, and BDC1 and BDC3 as a function of age. There was a correlation in which the younger astronauts tended to have a larger reduction in visual weighting associated with exposure to space. This could imply that younger astronauts are more flexible in adjusting to novel environments, or that they are more vulnerable to visual degradation in space.

### Astronauts and ground control participants

Astronauts are specially chosen and meticulously trained individuals. It might then be supposed that astronaut performance on ground tasks—especially during BDC1—would be superior to our naïve ground control group. As a consequence, one might have expected that BDC1 variances for astronauts would be significantly less than those reported for ground control participants. Such an effect was not found here, and a Mann Whitney U test indicated that there was also no significant difference between the baseline visual effects in the two groups (U = 23, *p* > 0.5). It would seem that having ‘The Right Stuff’ does not necessarily include being more precise in terms of estimating the direction of up.

## Conclusions

Knowing “which way is up” is fundamental to our survival. On earth, it is crucial to know where to put your feet to support your body and how to adjust to threats to this stability. In space, knowing which way is up is not needed for balance in the same way but is crucial for tasks such as knowing whether a toggle switch is in the on or off position and which way to go to get to the emergency hatch. On earth, gravity provides an effective and constant reference direction: a cue that is crucially absent in microgravity. Experiments using ground-based simulators including centrifuges, long-duration bed rest, water tanks, and microgravity aircraft^15^ have all addressed the question of how the perception of up is derived from the available cues and how it might be influenced by changes in gravity. Here, for the first time, it was possible to investigate the effects of long-duration microgravity on the process of perceiving the direction of up. We found that while the mean perceptual reports were not affected by spaceflight, the precision of judgments of the subjective visual vertical postflight was degraded.

The process of maintaining a perceptual upright such that it changes with visual cues in the expected way requires a fine rebalancing of the relative contribution of visual and body cues. What we have identified here is an exquisitely balanced system that responds well to even extreme changes that the human body could not possibly have evolved to cope with. The system adjusts by tweaking the relative sensory weights in response to the challenge of microgravity so as to ensure that vision continues to play a quantitatively similar role in space as it does on earth. However, these microgravity appropriate adjustments seem to persist for a long while after returning to gravity where they may not be as desirable.

## Materials and methods

### Participants

Seven astronauts volunteered to be a part of this experiment (mean age: 49, 2 female). The astronauts had various amounts of previous experience in space (three with none, three with 11–16 days, one with 193 days, see Table S1) and of course represent a highly trained and rigorously selected group. We used a control group on earth of 14 volunteers chosen to roughly balance the age and gender distribution of the astronauts (mean age: 41, 3 females). Due to the prolonged nature of this experiment (more than a year from start to finish) three of the control group were unable to complete the schedule and were dropped from the analysis. All experiments were approved by the appropriate ethics boards (see Supplemental Information for details).

### Equipment

All stimuli for both the astronauts and earth-bound observers, both on earth and in space were presented on a laptop display viewed through a short circular tunnel known as the COGNI tunnel, to remove other visual cues (see Fig. S1). Responses were collected by means of a game pad integrated with the COGNI tunnel. For the on-earth trials observers viewed the displays upright or lying on their right side in which case the display was tilted with them so that 0° always corresponded to the top of their head. For the in-space trials the COGNI tunnel was fixed to the wall of the spacecraft using a Bogan arm and the astronauts held onto the gamepad, which was firmly attached to right side of the COGNI tunnel. Thus, although care was taken that astronaut observers were free floating on the ISS in the sense that their body was not touching any reference surface, they were anchored to the ISS by virtue of holding on to the COGNI tunnel attached to the wall of the space station. While in space, observers were instructed to view the screen always from the “conventional orientation” which was clear because of the asymmetry of the COGNI tunnel’s viewing mask.

### Stimuli

#### The SVV

The subjective visual vertical was measured by means of a line superimposed on a background (Fig. S2a). The line measured 6° × 1° and radiated out from a dot (dia 1°) in the center of the screen (see Table S3 for details). The observer was required to judge whether the line was tilted to the left or right of gravity (the direction in which a ball would fall), a forced-choice recognition task, and to respond with button presses on the gamepad. The probe was presented for 500 ms and then replaced with a gray background of the same mean luminous with a fixation marker. The gray screen remained until the observer responded.

#### PU

The perceptual upright is defined as the orientation at which objects are most accurately and quickly identified.^37,38^ To find this orientation we exploited the fact that the identity of some shapes depends on their orientation. We used the ambiguous character “p” which appears as a “d” when rotated by 180°. The character measured 6° × 4° and was viewed at a distance of 8.25”. The probe was presented at one of 24 orientations (see Table S3 for details) and the participant’s task was to indicate whether the shape appeared to be a “p” or a “d” (a forced-choice recognition task). As for the SVV, the probe was viewed for 500 ms and then replaced with the gray background and fixation marker, which remained on until the observer responded (Fig. S2b). The orientations at which the character appeared most ambiguous were determined by fitting a psychometric function (see Supplemental Materials). The perceptual upright was defined as the orientation midway between these two most-ambiguous orientations.

### Procedure

#### Timing

The NASA convention is to refer to all experiments performed on earth as “BDC” sessions. We follow this convention here and refer to three BDC sessions corresponding to before launch (BDC1, average 107 days before launch), as soon as possible after return (BDC2, average 12 days after return), and later after return (BDC3, average 130 days after return). Measurements obtained on orbit and at the approximately matching times in our control group are referred to as “flight data” (FLT), which were either early in flight (FLTE, average 10 days after launch), late in flight (FLTL, average 18 days before return), or as part of an ordered sequence (FLT1, FLT2, etc). Although the astronauts had committed to run in-flight experiments at least twice (once early and once late in the flight), astronauts A–D were also able to run additional sessions. We did not attempt to mimic these additional trial times on earth but instead ran all control participants for five simulated in-flight sessions. The timings of all the sessions that make up this study are shown in Table S2.

#### Measurements

In order to evaluate the relative contributions of each cue, we separated the directions that each one indicated. On earth, before and after spaceflight, this was achieved by laying the observer on their right side so that gravity and body cues were orthogonal. The visual cue was controlled by presenting the probe superimposed on a photograph with clear orientation cues that could be tilted left or right. Other visual cues were obscured by viewing through the tunnel of the COGNI tunnel (see Supplemental Materials for more details). The in-space experiments were performed free floating with no part of the astronaut’s body touching any surface except the COGNI tunnel itself. The direction of the visual cue was altered relative to the body using the same display hardware and stimuli as used for the ground-based experiments. Each BDC consisted of four blocks, each block using either the PU or the SVV probe with the body upright or right side down. The blocks were run in a counterbalanced order. For each body orientation the line probe (SVV) was presented in various orientations superimposed on one of four backgrounds (gray, aligned with the body, or tilted left or right with respect to the body). For the PU, the same body orientations and visual backgrounds were used on earth, with the in-flight trials run free floating. No instructions regarding the background were given. Each probe orientation/background combination was presented seven times in a random order for each body orientation. The conditions are summarized in Table S3. Each condition took about 15 min to run. Prior to BDC1 all astronauts and controls participated in a brief training session.

#### Data analysis

The SVV and PU were assessed from psychometric functions (see Supplemental Material for details), which allowed us also to measure the variance associated with each measurement. Variance is defined as the square of the standard deviation of the responses and is a measure of reliability: a larger variance indicating a less reliable judgment. Values were averaged across all participants and are reported with the corresponding standard errors. When upright without a visual cue, the variance was due only to the gravity and body cues (or just the body cue in the case of a free-floating astronaut). Adding a visual cue therefore allowed us to assess the effect of vision on the variance. Thus, we could monitor whether any changes in the weighting of vision was a consequence of changes in the reliability of the visual cue, in accordance with maximum likelihood estimation theory which states that in multisensory integration cues are weighted inversely proportional to their variance (with certain assumptions).^1,39^ Repeated measures ANOVAs were performed on the PU and SVV values and on their associated variances. If Mauchly’s test for sphericity was violated for a given analysis then degrees of freedom were corrected using Greenhouse-Greisser.

## Electronic supplementary material

Supplemental material
