# Supplementary material for: The effect of long-term exposure to microgravity on the perception of upright
Source: NPJ Microgravity. 2017 Jan 12;3:3. doi: 10.1038/s41526-016-0005-5 (PMC5445609; doi:10.1038/s41526-016-0005-5)
Supplement: Supplementary file 1 — Supplemental material [file 41526_2016_5_MOESM1_ESM.docx]

**Supplemental information for:**

**The effect of long-term exposure to microgravity on the perception of upright**

**Laurence R Harris, Michael Jenkin, Heather Jenkin, James E. Zacher, Richard T. Dyde**

Here we provide details of the participants who took part in this experiment (table S1) , the timing of their data collection sessions (table S2) and the details of which conditions were tested (table S3) .

Photographs of the equipment used and the configurations in which it was used are provided in Figure S1. Figure S2 shows the timing of the stimulus presentations.

*Data analysis*

*The subjective visual vertical (SVV)*

For each background, responses were plotted as the frequency with which participants chose: “tilted left”. Examples of these plots are given Figure S3a. The data were fit with a hyperbolic tangent in polar coordinates (a shifted and scaled version of the sigmoid function). These fits measured not only the orientation of the SVV (x_0_) but also gave an estimate of the difficulty of the task in terms of t. The standard deviation (σ) of the measures was computed from t. Variance is the square of the standard deviation (σ^2^).

fit(x; x_0_, t) = 0.5 * (1+tanh((x-x_0_)/t))……………………………………………(1)

*Perceptual Upright (OCHART)*

The orientation of the PU was determined by fitting a double psychometric function to the frequency with which they chose “p”. Examples of these curves are given in Figure S3b. The data were fit with a product of two hyperbolic tangents in polar coordinates with a common scale factor (t). The PU was defined as half way between the two PSEs (x_0_ and x_1_). As with SVV, the standard deviation (σ) of the measures was computed from t.

Fit(x; x_0_,x_1_,t) = 0.5 * (1 – tanh((x-x_0_)/t) * tanh((x-x_1_)/t))……………………(2)

*Model fitting process.*

In this paper we modeled the data using a linear weighted vector sum model. Although the model is linear in terms of weighting and vectors, the probe responses are angles: combining these directions and angular measurements results in a non-linear optimization process. The three-vector model was therefore fitted using a non-linear least-squares optimization for each probe-body orientation condition using Python’s SciPy minimization function configured to use the Broyden – Fletcher – Goldfarb – Shanno algorithm. The data shown in Figure 4 are the mean of these weights across participants.

*Limitations of the linear weighted vector sum model*

The linear weighted vector model makes a number of simplifying assumptions (e.g., no impact of torsional eye roll ^1,2^, non-linear effects of tilt on the otoliths^3^ ). It is difficult to quantify the magnitude of the effect of these simplifications given the constraints on amount of data that we were able to collect. A related issue is the assumptions underlying the Bayesian integration of independent measurements of a single event ^4^ . Again more sophisticated models are possible. Further experiments are required in order to explore both of these directions.

|  |  | *gender* | *age at first data collection* | *prior space experience (days)* |
| --- | --- | --- | --- | --- |
| Astronauts | A | Female | 47 | 0 |
|  | B | Male | 48 | 11 |
|  | C | Male | 56 | 16 |
|  | D | Male | 50 | 0 |
|  | E | Male | 51 | 193 |
|  | F | Male | 50 | 0 |
|  | G | Female | 41 | 13 |
| Controls | g01 | Female | 50 |  |
|  | g02 | Male | 26 |  |
|  | g03 | Male | 50 |  |
|  | g04 | Male | 50 |  |
|  | g05 | Female | 38 |  |
|  | g08 | Male | 27 |  |
|  | g09 | Male | 45 |  |
|  | g10 | Male | 25 |  |
|  | g12 | Male | 55 |  |
|  | g13 | Female | 35 |  |
|  | g14 | Male | 45 |  |

*Table S1. Information about the participants in this experiment. None of the control group had any prior experience with our tasks. Participants had normal, or corrected-to-normal vision. No participant reported any history of vestibular disease or impairment. Experiments were approved by the York University Research Ethics Board Committee, the Canadian Space Agency and conformed to the requirement of the agreements for NASA/JSC Human Research Informed Consent, Multinational Human Research Informed Consent, and NASA/JSC Human Research Informed Consent for Grants.*


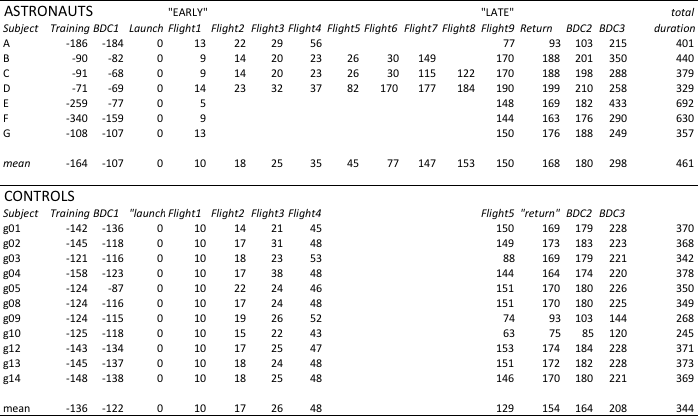


*Table S2 shows the timings of all the experiments presented in this study. The day of data collection is shown relative to launch date. For the controls this was defined as 10 days prior to the “flight 1” data collection date to match the average duration of this gaps for the astronauts. “Return” for the control group was taken as 10 days before the BDC2 data collection session. The astronauts committed to run the experiment at least twice in space. Astronauts A-D however ran many additional sessions as shown in the table. Data from “training sessions” are not included in the analysis.*

|  | Body orientation | Visual background | Test | Probe orientations | Number of trails (7 repeats) |
| --- | --- | --- | --- | --- | --- |
| ON EARTH | Upright | Grey, upright, 112° left, 112° right | PU | From -180° to +180° in 15° steps | 24 x 4 x 7 = 672 |
|  |  |  | SVV | From -55° to +55° in 5° steps | 22 x 4 x 7 = 616 |
|  | Right side down | Grey, upright, 112° left, 112° right | PU | From -180° to +180° in 15° steps | 24 x 4 x 7 = 672 |
|  |  |  | SVV | From -135° to -45° in 5° steps | 36 x 4 x 7 = 1,008 |
| IN FLIGHT | Free floating | Grey, upright, 112° left, 112° right | PU | From -180° to +180° in 15° steps | 24 x 4 x7 = 672 |

*Table S3. Showing the conditions tested. PU=perceptual upright, SVV= subjective visual vertical. For the on-ground controls, the “free floating” on-orbit tests were paralleled with same PU tests as the astronauts, but performed upright.*

*
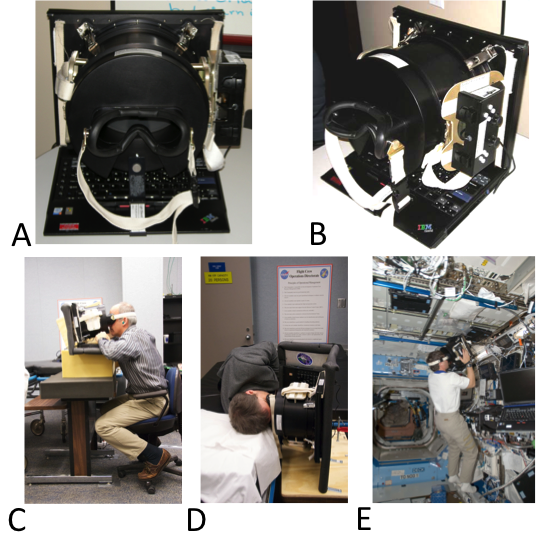
*

*Figure S1. A, B The COGNI Tunnel. This device consisted of an IBM GSE T61p laptop integrated with a fixed display tunnel and input panel. Participants viewed the display through a circular aperture (dia. 9.25” viewing angle 60°), which also served to control their viewing distance at 8.25”. The screen had a resolution of 1024x768 pixels. A diffuser set across the far end of the tube, against the display screen, blurred the screen enough to render the pixels invisible. This was the display device used for ground-based data collection either upright (C) or with both the observer and the tunnel tilted by 90°(D). In space it was attached to the wall of the International Space Station (E). E is a NASA image.*


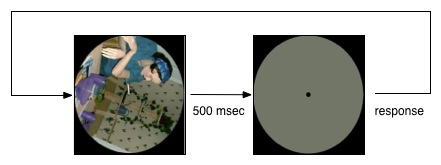


(A) Subjective visual vertical (SVV)


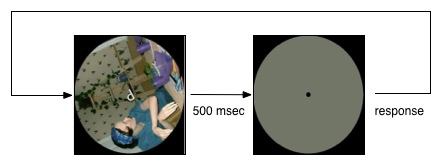


(B) Perceptual upright (PU)

*Figure S2, The experimental protocol.*

*
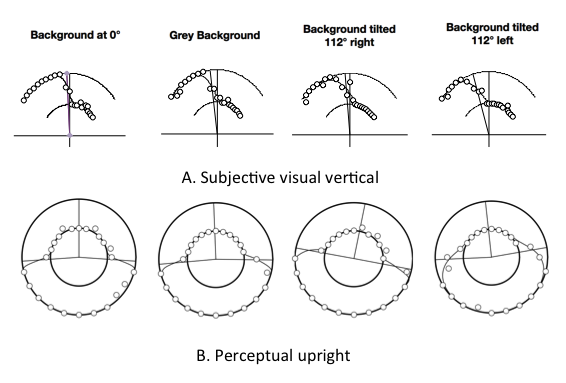
*

*Figure S3. Examples of participant response frequencies for SVV (A) and PU (B). Each row shows responses for one participant for the four backgrounds for a single body orientation. (A) SVV: A psychometric function is plotted through the data in polar coordinates where the outer circle represents 100% “probe tilted to the left of gravity” and the inner circle represents 100% “probe tilted to the right of gravity”. The SVV is the 50% point of this curve. (B) PU: The product of two psychometric functions is plotted through the data in polar coordinates where the outer circle represents 100% “probe interpreted as a ‘d’” and the inner circle represents 100% “probe interpreted as a ‘p’”. The PU is defined as the midpoint between the 50% points of the two psychometric functions, indicated by radial lines on the figure.*

SUPPLEMENTAL REFERENCES

1 Bockisch CJ, Haslwanter T. Three-dimensional eye position during static roll and pitch in humans. *Vision Res* 2001; **41**: 2127–37.

2 Wade SW, Curthoys IS. The effect of ocular torsional position on perception of the roll-tilt of visual stimuli. *Vis Res* 1997.

3 Mittelstaedt H. The Role of the Otoliths in Perception of the Vertical and in Path Integration. *Ann NY Acad Sci* 1999; **871**: 334–344.

4 Ernst MO. Optimal multisensory integration: Assumptions and limits. In: Stein B (ed). *The New Handbook of Multisensory Processing*. MIT Press: Cambridge, Mass, 2012, pp 527–544.
